# Semaglutide and the Retina

**DOI:** 10.1111/1753-0407.70085

**Published:** 2025-04-14

**Authors:** Zachary T. Bloomgarden

**Affiliations:** ^1^ Division of Endocrinology, Diabetes and Bone Disease, Department of Medicine Icahn School of Medicine at Mount Sinai New York New York USA

**Keywords:** diabetes, glucagon‐like peptide‐1 receptor agonists, retinopathy

## 1 Introduction

Globally, diabetic retinopathy (DR) is estimated to have affected 103 persons in 2020, with projections of 130 and 161 million in 2030 and 2045, respectively. The respective prevalences of vision‐threatening DR, are 29, 36, and 45 million persons in 2020, 2030, and 2045, and of clinically significant diabetic macular edema (DME), 19, 24, and 29 million persons [1]. Glucagon‐like peptide‐1 Receptor Activators (GLP‐1RA) have become more widely used in the treatment of type 2 diabetes (T2D), with analysis of the TriNetX dataset showing that the prevalence of GLP‐1RA use in the US increased from 6.4% in 2018 to 14.9% in 2022 among T2D with ASCVD [2]. 12% of US adults say they have taken a GLP‐1 agonist, and 6% say they are taking this currently; the prevalence of such use is 43% among people with diabetes, 25% among those who have been told of heart disease, and 22% among those who have been told by a doctor about being overweight or obese [3].

We generally would anticipate excellent glycemic treatment to be associated with improved outcomes of most diabetes complications. Indeed, a meta‐analysis of 11 randomized controlled trials (RCT) involving 51 469 patients with T2D showed a 15% reduction in DR risk [4]. The GLP‐1RA are among the most effective agents used in T2D treatment, having at least as great a glycemic effect as basal insulin [5], with weight loss rather than weight gain and with a lower likelihood of hypoglycemia. A caveat is the recognized potential that with rapid improvement of poorly controlled diabetes conditions similar to the microvascular complications of diabetes can occur. Acute painful peripheral diabetic neuropathy is one such syndrome [6]. Another such condition was seen in the analysis of the initial outcome of the Diabetes Complications and Control Trial (DCCT) of type 1 diabetes, in which early worsening of DR was observed at the 6‐ and 12‐month DCCT visits in 13.1% of 711 patients assigned to intensive treatment and in 7.6% of 728 patients assigned to conventional treatment, particularly in those with higher baseline HbA1c and in those with a greater 6‐month reduction in HbA1c [7].

## 2 Effect of GLP‐1RA on DR: Population Studies

The effect of GLP‐1RA on DR is less clear. Using the TriNetX global research network to assess the development of DR and DME in ~2 million individuals with type 2 diabetes taking insulin, one study using propensity score matching suggested that those receiving GLP‐1RA had a 31% increased risk of DR, without significant change in the risk of DME, compared with those receiving neither sodium‐glucose co‐transporter 2 inhibitors (SGLT2i) nor GLP‐1RA, while compared with those receiving SGLT2i, those receiving GLP‐1RA had a 20% higher risk of DR and a 13% higher risk of DME [8]. However, another study using TriNetX among 9.9 million patients with T2D, over a 2‐year period of observation, found that DME was 23% and 17% less likely with GLP‐1RA [9]. In the Taiwan National Health Insurance Research Database, of 97 413 patients initiating either GLP‐1RA or SGLT2i in 2016–2017, propensity score matching of those with a previous dx of diabetic retinopathy showed a 50% increase in risk of progression with GLP‐1RA, primarily related to tractional retinal detachment; of those without previous history of diabetic retinopathy, ocular outcomes were similar with the two agents [10]. Using data from the Danish National Patient Registry of T2D, among patients not taking insulin, metformin + GLP‐1‐RA was associated with a 1.46‐fold increased risk of diabetic retinopathy compared with metformin + dipeptidyl peptidase‐4 inhibitors (DPP‐4i), with metformin + SGLT2i trending to still lower risk [11]. Other, smaller studies do not show an increase in the likelihood of DR with GLP‐1RA [12]. The FDA adverse event reporting system (FAERS) has been widely criticized as being subject to ascertainment bias, but an analysis using this system reported 5003 ophthalmic adverse events (AE) associated with GLP‐1 RA from 2018 to 2023, particularly for semaglutide, liraglutide, and exenatide, peaking in 2021, with an increasing percentage associated with semaglutide from 2021 to 2023 [13].

## 3 The SUSTAIN‐6 Trial

In pre‐cardiovascular outcome trials (CVOT) of semaglutide, baseline history of diabetic retinopathy was present in 3.7%–14.5% of participants, and retinopathy AEs developed in 2.1% and 1.5% of participants treated with semaglutide 0.5 and 1.0 mg weekly versus 2.0% of comparators, a reassuring finding. However, the Semaglutide Unabated Sustainability in Treatment of Type 2 Diabetes (SUSTAIN)‐6 CVOT of 3297 persons with T2D having high cardiovascular risk [14] included 29.4% of participants with a history of retinopathy, and retinopathy AEs developed in 9.0% and 10.0% of participants treated with semaglutide 0.5 and 1.0 mg weekly, respectively, but significantly less frequently in 7.6% of comparators. In patients with pre‐existing DR at baseline, the risk of DR worsening was further increased among patients treated with insulin; both among those receiving semaglutide and among those receiving placebo, DR worsening occurred most often among participants having HbA1c reduction > 1.5% [15]. The question then is whether GLP‐1RA in general, and semaglutide in particular, might have a specific adverse effect on diabetic retinopathy, or whether the marked efficacy of these agents in improving glycemia might simply be associated with acute worsening of DR.

## 4 Differing Results of Meta‐Analyses

A large number of meta‐analyses have been undertaken to ascertain whether GLP‐1RA have specific adverse effects on DR, or whether this is simply the expected finding with marked improvement in glycemic control.

Four such studies concluded that on balance there was little evidence of specific adverse effects of GLP‐1RA. A meta‐analysis of 61 RCTs involving a total of 188 463 patients and 2773 DR incidents, including 29, 13, and 10 studies of GLP‐1RA, DPP4i, and SGLT2i versus placebo, respectively showed no significant difference in DR, as did 8 studies of GLP‐1RA versus DPP4i, leading to the conclusion that “[these do not] differ significantly in their effect on diabetic retinopathy complications.” [16] In a meta‐analysis of 20 RCTs of 24 832 persons with T2D treated with GLP‐1RA versus placebo, no significant effect was seen on development of diabetic retinopathy (Odds Ratio (OR) = 1.17, 95% CI: 0.98–1.39, *p* = 0.25), with subgroup analysis by agent showing similar effects of liraglutide (OR = 0.86, 0.50–1.49), parenteral semaglutide (OR = 1.12, 0.67–1.86), lixisenatide (1.5, 0.06–37.08), albiglutide (1.02, 0.77–1.35), and efpegenatide (1.69, 0.08–35.58), although there was significant increase in DR with oral semagutide (OR = 1.43, 1.09–1.87) [17]. In another analysis of all GLP‐1RA CVOT with information about retinopathy (mean trial duration 3.1 years), and all semaglutide RCT with such information (mean trial duration 1.3 years), worsening retinopathy only occurred with semaglutide, but was found to occur in proportion to the decrease in A1c, with the increase in retinopathy for semaglutide associated with therapy duration > 1 year and with HbA1c decrease > 1% [18]. Finally, among six RCTs of 49 936 persons with T2D randomized to GLP‐1RA versus placebo which included retinopathy as a prespecified end point, meta‐analysis showed no significant association between GLP‐1RA and retinopathy risk (OR 1.10; 95% CI 0.93, 1.30), while meta‐regression analysis showed a significant association of retinopathy with greater average reduction in HbA1c [19].

In contrast, three studies found GLP‐1RA to be associated with adverse DR outcomes. A meta‐analysis of 93 RCT of GLP‐1RA compared with insulin, oral agents, or placebo in 106 819 participants found that those participants randomized to GLP‐1 RA had a 31% greater risk of early‐stage DR compared to placebo; although, compared to insulin, GLP‐1 RA use was associated with a 62% lower risk of late‐stage DR [20]. A meta‐analysis of seven cohort studies including 242 537 patients with type 2 diabetes showed that those receiving GLP‐1RA had a 34% lower incidence of DR than those treated with insulin; although DR complications such as vitreous hemorrhage, retinal detachment, or need for treatment with intravitreal injections, lasers, or vitrectomy occurred with similar frequency among both groups; comparing GLP‐1RA with oral antidiabetic agents, there was a similar incidence of DR, but GLP‐1RA were associated with a 39% greater risk of DR complications and a 40% greater likelihood of progression to proliferative DR [21]. Finally, a meta‐analysis of 23 RCTs of semaglutide including 22 096 patients with T2D with 730 incident DR cases showed a relative risk (RR) of DR of 1.14 (95% CI 0.98–1.33); but, compared with placebo, the RR was 1.24 (1.03–1.50); with patient age ≥ 60 the RR was 1.27 (1.02–1.59), and with diabetes duration ≥ 10 years the RR was 1.28 (1.04–1.5) [22].

## 5 Nonarteritic Anterior Ischemic Optic Neuropathy

Non‐Arteritic Ischemic Optic Neuropathy (NAION) is a condition of ischemic infarction of the optic nerve head manifesting as unilateral optic disc edema and abrupt painless vision loss, typically in individuals with high CV risk [23]. A recent case series described NAION and related complications in four persons after receiving semaglutide and in three receiving tirzepatide [24]. In the 16 827 person database of individuals who had been referred for neuro‐ophthalmological assessment at Massachusetts Eye and Ear with no history of NAION, 710 had T2D, of whom 194 were prescribed semaglutide and 516 prescribed non‐GLP‐1 RA antidiabetic medications; 17 NAION events occurred in patients prescribed semaglutide versus 6 in the non‐GLP‐1 RA cohort, with cumulative incidence of NAION over 36 months for the semaglutide versus non‐GLP‐1 RA cohorts 8.9% versus 1.8%. In addition to the group with T2D, 979 were overweight or obese, of whom 361 were prescribed semaglutide and 618 prescribed non‐GLP‐1 RA weight‐loss medications, with 20 NAION events in those prescribed semaglutide versus 3 in the non‐GLP‐1 RA cohort, for 36‐month incidence of NAION for the semaglutide versus non‐GLP‐1 RA cohorts 6.7% and 0.8%. The GLP‐1RA versus other medication groups were compared with propensity score matching against age, sex, hypertension, hyperlipidemia, T2D, obstructive sleep apnea, and coronary artery disease, showing Hazard Ratios (HR) of 4.28 and 7.64 in the diabetes and non‐diabetes comparison sets, respectively [25]. In a 5‐year longitudinal cohort study of all 424 152 persons with T2D in Denmark between 2018 and 2024, 106 454 were treated with once‐weekly semaglutide versus 317 698 not so treated. 67 versus 151 developed NAION, with onset of NAION evenly distributed within the entire 5‐year observation period, for significantly higher incidence rates of 0.228 versus 0.093 per 1000 person‐years and a hazard ratio (HR) of 2.19 adjusted for sex, age, marital status, duration of diabetes, hemoglobin A1c, estimated glomerular filtration rate, cardiovascular disease, use of insulin, use of cholesterol lowering medicine, and use of blood pressure lowering medicines. In the multivariable model the HR for semaglutide was 2.42 and 2.62 for once weekly semaglutide without and with SGLT2 inhibitors, and 2.26 for persons without previously diagnosed diabetic retinopathy [26]. Data from national health registries in Denmark and Norway identified 16 860 new users of semaglutide during 2018–2022 in Norway with 8 NAION events, and 44 517 new users during 2018–2024 in Denmark with 24 NAION events; compared with sodium‐glucose co‐transporter 2 inhibitor (SGLT‐2i) users the pooled HR was 2.81 with a greater incidence rate by 1.41 episodes per 10 000 person‐years. During 1‐year follow‐up in Denmark, less than five NAION events were seen among users of semaglutide for the treatment of obesity without diabetes [27]. One population‐based study using the TriNetX database compared persons treated with semaglutide versus propensity score matched persons not receiving GLP‐1RA among 37 314 persons with T2D with BMI < 30, 129 690 with T2D having BMI > 30, and 129 690 with BMI > 30 not having diabetes, without significantly increased risk of NAION [28], and an analysis using propensity score matching to query two clinical databases also failed to show such an effect of semaglutide or any other GLP‐1RA [29]. However, another analysis using the TriNetX database showed that, although a significant effect of GLP‐1RA was not seen earlier, there was more than a doubling in risk of NAION after 2, 3, and 4 years of treatment [30].

## 6 Implications

We are left with two potential explanations of these findings. First, GLP‐1RA are associated with great and often rapid improvement in glycemia, which may lead to DR, to DR progression, or, less commonly, to NAION. Second, semaglutide may be separately associated with these ophthalmologic complications. It should be noted that, although nine RCTs with a total of 10 164 patients with T2D, including 6700 receiving tirzepatide and 3464 receiving control treatment, showed no effect on diabetic retinopathy risk at any dose, patients were excluded for proliferative DR, for diabetic macular edema, and for nonproliferative DR requiring treatment [31]; studies in susceptible individuals are needed to ascertain whether semaglutide is associated with a different magnitude of risk from that with other potent incretin‐based agents. The authors of the Scandinavian study comment that their observed finding of the rate of NAION in semaglutide‐treated persons with type 2 diabetes corresponds to an absolute risk of 0.3%–0.5% over 20 years, and as such does not negate the numerically far greater CV/renal benefit of this treatment.

What is appropriate in patients for whom semaglutide is indicated based on high HbA1c, obesity, and risk of cardiac and renal disease, particularly among those with DR? Should intraocular vascular endothelial growth factor antagonist treatment be considered in patients with advanced nonproliferative DR when starting GLP‐1RA [32]? Population‐based studies to allow us to assess retinal along with glycemic, weight loss, and CV/renal effects will be of great importance.

## Conflicts of Interest

The author declares no conflicts of interest.
